# Home-Living Elderly People's Views on Food and Meals

**DOI:** 10.1155/2012/761291

**Published:** 2012-09-09

**Authors:** Ellinor Edfors, Albert Westergren

**Affiliations:** The PRO-CARE Group, School of Health and Society, Kristianstad University, 291 88 Kristianstad, Sweden

## Abstract

*Background*. The aim of the study was to describe home-living elderly people's views on the importance of food and meals. *Methods*. Semistructured interviews with twelve elderly people. The interviews were analysed using qualitative content analysis. *Results*. Respondents described how their past influenced their present experiences and views on food and meals. Increased reliance on and need of support with food and meals frequently arose in connection with major changes in their life situations. Sudden events meant a breaking point with a transition from independence to dependence and a need for assistance from relatives and/or the community. With the perspective from the past and in the context of dependency, respondents described meals during the day, quality of food, buying, transporting, cooking, and eating food. *Conclusions*. Meeting the need for optimal nutritional status for older people living at home requires knowledge of individual preferences and habits, from both their earlier and current lives. It is important to pay attention to risk factors that could compromise an individual's ability to independently manage their diet, such as major life events and hospitalisation. Individual needs for self-determination and involvement should be considered in planning and development efforts for elderly people related to food and meals.

## 1. Introduction

Malnutrition, which includes undernutrition as well as overweight/obesity, is a common problem among the elderly. The prevalence of undernutrition among home-living elderly people was found to be 14.5% according to the Mini Nutritional Assessment (MNA) [1]. Among elderly people who had recently moved to a residential home, 33–37% were malnourished according to the MNA [2]. The higher prevalence of undernutrition among elderly people admitted to residential homes highlights the importance of identifying elderly people who live at home and are at risk of malnutrition, to prevent the development and aggravation of undernutrition, followed by increased dependency on and need for institutional care [1]. In addition, among 70-year-old Swedes, 20% of the men and 24% of the women were obese (BMI > 30) [3]. In Swedish nursing homes the prevalence of overweight was 22% and another 8% were obese [4]. Thus, obesity is also a frequent problem among the elderly.

The consequences of undernutrition in elderly people include functional decline or frailty [5–7], decreased quality of life [8], increased health care utilisation and costs [9, 10], higher rates of adverse complications from other health conditions [11], and increased mortality [6, 7, 12]. The consequences of obesity include negative impact on physical functions and quality of life, decreased survival rates, metabolic syndromes, arthritis, pulmonary abnormalities, urinary incontinence, cataracts, and cancer [13]. Thus, from a nutritional perspective, obesity as well as undernutrition are a concern among elderly people as they exacerbate age-related decline in physical functioning and can cause frailty [13].

Myriad risk factors are associated with inadequate caloric intake and malnutrition. Risk factors for undernutrition have been identified as higher age, lower self-perceived health, low functional status, diseases, taking several medicines, and symptoms of depression [1, 14–18]. It was also found that people who were hospitalised prior to receiving home health services were more likely to undereat than those not hospitalised [19]. Eating with others increases caloric intake [20]. Aging is associated with a decrease in total energy expenditure, and if this coincides with a maintained or increased energy intake, overweight/obesity may develop [13]. 

Currently in Sweden, fewer elderly people with functional impairment move to nursing homes, and more choose or have to remain in their own homes. Many of these people are disabled and dependent on others for acquiring, preparing and/or consuming their food. The need for help with acquiring food typically occurs before the need for assistance with meal preparation arises [21]. Some elderly people get help with these activities from informal providers. Previous research has shown that living with others or receiving help from informal providers is beneficial for dietary intake [22, 23], and that frequent provision of meals from a formal agency can improve food intake [24] and decrease undernutrition risk [21]. Furthermore, by getting meals from a formal agency, those who would otherwise not be able to obtain or prepare food without assistance are able to keep living in their own homes [25].

Considering all the negative effects of malnutrition, it seems of utmost importance to identify elderly people living at home at risk of developing malnutrition, or who have already developed it, so that suitable preventive actions and/or treatment can be provided. To do so we need a deeper understanding of the circumstances of older people living at home, with respect to nutrition, from their perspectives. The aim of the study was to describe home-living elderly people's views on circumstances that are of importance regarding food and meals.

## 2. Methodology

The study was carried out in a small community in southern Sweden (approximately 12500 inhabitants), containing two smaller towns. Elderly care interventions, such as getting meals from a formal agency (food distribution/meals on wheels), were provided from two different kitchens, one localised in the towns. The meals were distributed to the elderly by home care staff. The elderly person had to pay a subsidised fee for the meal and meal delivery. Even though respondents lived independently they could have access to elderly centres for social activities and restaurants.

This study was a descriptive qualitative study based on semistructured interviews with twelve elderly people living in their own homes in a small municipality in southern Sweden. Criteria for inclusion in the study were elderly people over age 65 living in their own homes (with or without home help service and/or meals from a formal agency) and able to communicate in Swedish. Respondents were recruited by the nutritionally responsible nurse and the unit managers in the municipality. Written information about the aim of the study, that participation was voluntary and who to contact for further information, was distributed to potential respondents by home care staff. It is unknown how many people received this information. Those who agreed to participate were contacted by phone by the first author (E. Edfors); further information about the study was given and the time and place for the interview were set. 

Twelve people were interested and actually participated in the study, seven men and five women, aged 82 to 94 (average 87.7 years). Four of them lived as couples and both partners were included in the study. Six of the respondents had no home help service and out of these three had food distribution from a formal agency. Three of the respondents had no food distribution while nine had food distribution that varied from three to seven days a week. Half of the respondents rated their severity of disease as moderate and only one as severe (Table 1).

The interviews were conducted by the first author (EE) in the respondents' homes. Before the interview started, the respondent was given clarifying information about the aim of the study, their right to withdraw at any time with no personal consequences, and that participation was voluntary. Written informed consent was obtained. As there could be a risk that the interviews were perceived as an invasion of privacy and cause emotional strain, respondents were offered the opportunity to contact the authors afterwards. All collected materials and personal data relating to respondents were treated confidentially. The study was performed in accordance with the Helsinki declaration of ethical principles [26]. Formal approval was not needed for this type of study, in accordance with Swedish law [27].

The interviews were conducted as semistructured interviews [28] based on the aim of the study. The interview started with an open-ended question and the respondent was asked to talk freely about an ordinary day, focusing on food and meals, “describe an ordinary day and try to focus on the food and meals during the day.” Thereafter the questions were about food preferences and intake, physiological difficulties (swallowing, chewing), and functional difficulties (cooking, shopping), and on social dimensions of eating. These questions were inspired from themes in “Seniors in the Community: Risk Evaluation for Eating and Nutrition, Version II” (SCREEN II) that measures the risk of malnutrition among elderly people living in their own home [29]. Throughout the interview, the author, using follow-up questions, tried to capture the importance of circumstances and factors affecting food and meals. The interviews lasted 40–90 minutes and were recorded with digital equipment and transcribed verbatim.

The interview texts were inductively analysed by using qualitative content analysis [30]. Content analysis is described on two levels: the manifest content analysis that focuses on the content of the texts from a superficial perspective based on the written word, and latent content analysis that goes in-depth on content and interprets the underlying meaning conveyed by the text [30]. In this study the analysis was mainly based on manifest content analysis. The analysis was conducted in all stages by the two authors and work continuously alternated between the whole and parts of the collected material. In step one, texts were read and reread as a whole, a so-called naive reading. Statements were then made based on the impressions of and reflections about the wholeness and important elements in the text that had emerged during the naive reading. Then all parts of the text relating to the aim of the study were divided into meaning units that seemed to be about the same thing. In next step the meaning units were coded. The codes were critically discussed and a number of categories with subcategories emerged. Finally, all texts were re-read and compared with the outcome of the analysis to ensure that the categories covered the contents of the texts and codes. “Open Code” (freeware) software was used for the qualitative analysis (UMDAC and Epidemiology, University of Umeå).

## 3. Results

Three categories, with two-to-five subcategories each, were developed based on the text analysis: habits developed in the past affect the present; getting help from others with food and meals; current food and meals (Table 2).

### 3.1. Habits Founded in Past Life Affect Present Life

#### 3.1.1. Food and Meals

The interviews showed that experiences in childhood and earlier adulthood had a great impact on the respondent's current feelings and views concerning food and meals. It was evident that the foundations of norms and values regarding food culture, traditions and eating habits were laid early and did not change to any great extent throughout life.

* As children we were never allowed to say “I do not want that, I won't eat it.” No, that was just not possible (no. 4, female, age 94).*

*I eat the same type of food and about the same time as I always have done, as I did when my wife lived and when I worked (no. 9, male, age 89).*

The majority of the respondents had lived their lives in an environment where they were used to cooking for themselves and there was a difference between everyday meals and special occasions. Food and meals had also played an important social role. It was, for instance, important to be generous to the “outside world”.

* There were people in the whole house, and there were a lot of rooms, and there was food, a lot of food, yes really (no. 4, female, age 94).*

* I feel that when you are out, or come someplace, you should be offered a cup of coffee, that's what I'm used to. Back home in the country, the coffee would be on as soon as someone walked in the door (no. 4, female, age 94).*

The respondents had grown up on a diet of home-cooked dishes made from locally produced ingredients. Meals consisted of, for example, porridge, wholemeal bread, potatoes, pork, and fresh fish. At ceremonies and celebrations one might be offered freshly slaughtered meat and more luxurious food.

* I am used to home-cooked dishes since I was a child, not macaroni and spaghetti (no. 7, female, age 84).*

* “When I grew up, pigs were supposed to weigh 125 kg when they were slaughtered. There was a thick layer of fat on their backs” (no. 11, male, age 94).*

The diet was based on what the season had to offer and it was vital to “make do with what the house could offer” and seize every opportunity to increase the food supply, for instance, by hunting, fishing, and picking berries.

* I still pick berries and make syrup. I grew vegetables before (no. 10, female, age 88).*

* I grew up by the sea and do not know anything else. I have been fishing myself, and everything (no. 6, male, age 89).*

#### 3.1.2. Roles

The interviews showed a division in gender roles regarding the responsibility for the diet. Usually, the woman in the family had the main responsibility for food and meals. *“My wife was a very good cook” *(no. 5, male, age 82). The majority said that they had always been served homemade and carefully cooked food and that they knew how good and nutritious food should taste.

* I worked as a cook; I used to do the cooking at home before I got my heart attack (no. 1, male, age 84).*

* I've always been a good cook, and have always appreciated good food. My husband always said, “you cook such good food, I get way too fat”. But I so appreciated good things…. I wanted… if you are going to eat, you might as well have good food (no. 4, female, age 94).*

### 3.2. To Get Help from Others with Food and Meals

#### 3.2.1. The Breaking Point

The interviews revealed that in several cases, major changes in the ability to be independently responsible for food and meals were linked to some form of sudden event. It could be that the female partner who was responsible for meals passed away. Other causes could be falls, infections, and other diseases, such as myocardial infarction, conditions that in many cases had led to a hospitalisation. Other factors that affected independence were general frailty and an inability *“to use one's old body*,*”* that is, factors linked to “normal ageing.”

* About five years ago my wife had an accident, and she could no longer do the cooking. We had to get food from the service house (no. 1, male, age 84).*

#### 3.2.2. Transition from Independence to Dependence

Becoming dependent on others on a daily basis can be difficult to deal with. One man had a strong desire to get well and return to independence.

* I believe that everything changes. I dream of being able to buy myself a car in the spring so that I can go shopping (no. 6, male, age 89).*

Other respondents felt that they had no choice but get used to it and accept the situation.

* I have become so used to it that I do not even think about whether it is difficult or not, it is just something you have to put up with (no. 4, female, age 94).*

One man seemed pleased to be dependent and he had strongly questioned the community's decision to discontinue his food distribution.

* Well now that it has been so long, you might consider cooking again (note, said aid assessors)… Oh, said I, aren't you being a bit too hard now… It's quite expensive, she said… will they lose five crowns? Ten crowns? I can pay the difference because I've never been a burden for the community. (no. 12, male, age 86).*

### 3.3. Food and Meals in Present Life

#### 3.3.1. Meals during the Day

Currently, and in the past, respondents' daily meals were distributed as breakfast, dinner and supper, usually served at the same time every day. The majority had their principal meal at noon, while supper mostly consisted of lighter food, such as tea and sandwiches. However, one male respondent preferred to have a proper cooked meal also for supper. From the interviews it could be discerned that the majority of respondents distributed their meals so that at night there was a long period without eating.

* All my life I've had lunch at twelve o clock. So I usually eat at noon, and then I eat once in the evening, usually very light food (no. 6, male, age 89).*

Regular snacks were not common, except when someone occasionally felt hungry and had, for example, a piece of fruit, a cookie, or a sandwich. On the other hand, snacks in the form of coffee breaks were considered a natural and important element in social gatherings with other people, such as when friends came for a visit or when the respondents participated in social activities.

* I do not eat snacks unless there is a study circle, like today, where we get served nice little cookie (no. 8, female, age 85).*

The four respondents who lived as couples ate their meals together, while single respondents mostly ate their meals alone. Two women felt lonesome with no companion at mealtimes. Four men, on the other hand, preferred to eat alone. They found it easier to be able to sit comfortably and eat at any time they wanted at their own pace, without having to consider other people.

* I think it's great, I have no major, no problems at all. I think it's nice to sit quietly and eat my meal (no. 1, male, age 84).*

Even though respondents lived independently they could have access to elderly centres for social activities and restaurants.

* I generally eat by myself. I have thought many times that I should go to the restaurant, because they do have a separate dining room at XXX (note, elderly centre). But I end up feeling more comfortable eating at home (no. 5, male, age 82).*

One woman said that eating at the elderly centre demanded suitable clothing, social behaviour and sociability. The noisy environment, caused by young people from a nearby school who also had their lunch there, could be another reason for eating alone at home instead.

* … And then the young people from the school there, they also eat there, and when they're there, it is not easy for the older people to be among them, since they are noisy and do not behave (no. 7, female, age 84).*

Overall, the interviews revealed that the respondents usually did not skip a meal. Their appetite was normally good. Poor appetite and weight loss could be caused by psychological malaise, other illnesses, grief, lack of outdoor activities and bad temper.

* Yes, it's psychological and that eliminates a lot of things. Very, very sensitive… when these problems occur (note, depression)… the food does not taste good, no. Then I ended up skipping meals and eating only two or three crackers a day (no. 11, male, age 94).*

Other reasons for a poor appetite or skipping a meal were related to the content of the distributed food, such as food having an unappealing appearance, not tasting good or containing ingredients that the older person did not like or tolerate, such as hot spices, or being difficult to chew and digest.

* And it is served in such an unappealing way in these trays, it's not exactly exciting. There is one container for the sauce and one for the potatoes and the pieces of meat just lying there… it's not, it does not give you an appetite… and you feel like, oh, that does not look good at all… (no. 4, female, age 94).*

#### 3.3.2. Quality of Food

The interviews revealed different perceptions of the quality of food among respondents who had food distribution. Respondents who got their food from a separate kitchen were very satisfied with the food content and quality. They understood that they would not always be served their favourite dishes. This group of respondents felt that the food consisted of varied and tasty dishes and appreciated the variation in content between weekday and weekend.

* Wonderful, yes… It is very, very, very good. I cannot complain… It's varied and that's good. There are some things I do not like so much, but I still do not cancel. I could… (no. 12, male, age 86).*

Other respondents, who got their food from another kitchen, felt that the food was poor, tasteless, and badly cooked. They also said the food looked unappetising, contained spices that they were not familiar with, and was too influenced by modern food trends, such as pizza. They requested more varied old fashioned food, cooked in the traditional way with well-known spices such as salt, pepper, dill, and bay leaves.

*It varies, it does, I'm being frank now… food for us elderly people is probably supposed to be cooked with care, but it happens that some food is not properly cooked” and “… It should be properly boiled or fried, depending on the dish. Very important. It happens that this is overcooked (no. 5, male, age 82).*

They also wanted certain ingredients in their food, such as fresh fish, veal, lamb, vegetables, fat, and cream. One respondent preferred the restaurant service and seemed to get much better food there than in food distribution. One woman chose to throw away food when it did not taste good or looked unappetising.

* Now that I have reached this age, I should be allowed to… have things I like, and not… food that makes me think: Ugh, what is that, that's no good. What do I need that for? I do not want that… straight into the rubbish… I've thrown out a lot of money there (no. 4, female, age 94).*

Usually it was the home care staff who received the complaints about the food, even though the respondents knew that they not could influence the food content to any significant degree. Several interviews revealed that respondents perceived shortcomings in the municipality's interest in listening to their views about the food distribution.

* I have talked to these girls. I feel sorry for the girls. They're the only ones I've talked to. The others, the people at the top, yes, well, I do not have time to talk to you right now, maybe I can call you some other time or something (no. 6, male, age 89).*

#### 3.3.3. Buying and Transporting Food

The interviews showed that those who lived in urban areas were satisfied with the availability of well-stocked supermarkets. Having more than one shop in the community was believed to promote choice, quality and good prices. The majority of the respondents emphasised the importance of having access to an open-air market that was open one day a week, which gave them the opportunity to buy local products and good quality fresh fish. An important service to one couple that lived outside the urban area was the regular visits of a fish van and a private supplier of food. 

One man and one woman, who arranged their shopping independently, said that the premise for this was that they could drive their car to the store themselves. Respondents who were no longer able to buy their food themselves stated different reasons for this, such as the inability to drive and difficulties in mobility. Five of the respondents, who got no help with shopping from the community, got help from children, other relatives and/or friends, usually once a week.

* I always keep a list. When I know that something is missing, I write it down, and then when he comes (note, the son) it's ready (no. 9, male, age 89).*

*Before we had four shops, now there is none. Now my daughter helps me with shopping (no. 12, male, age 86).*

One respondent, who had access to transport service (subsidised transport by taxi) for purchases, did not use this help because he tended to need more than one ride to and between the shops.

* I get transport service, but it's so complicated and difficult… I do not know quite how long I have, and sometimes I need to go to the pharmacy, and then I have to go all the way from the supermarket to the pharmacy, and then I get a pain in my chest and have to order an extra trip… (no. 1, male, age 84).*

Five of the respondents got community assistance with buying and transporting food, usually once a week. They had a strong desire to have control of food planning, such as shopping lists and purchases. Most of them wrote their own shopping list, and the shopping was carried out by the home care staff. Sometimes the respondent would forget to write things down on the list, or the wrong things were purchased by the home care staff. When this happened, the respondent usually had to wait until the next week's shopping.

* They bought the wrong things… That's why it's important that when I order something there are people who know what it is all about, especially those who have their own household. And then they send a girl working temporarily… Here I am … and sometimes when they are gone.. oh goodness, I forgot… (no. 6, male, age 89).*

One woman requested more than one hour for shopping, since the staff's short allotment of time for this purpose made it impossible for her to come along to the shop. She wanted to be able to compare what different shops had to offer and make her own decisions.

* One hour is too short. I want to spend some time in the shop. Two hours would give enough time to go to both XXX and YYY without hurry (note, two shops). See for yourself and not just request something. I do not know what they have and what's good. (no. 4, female, age 94).*

#### 3.3.4. Cooking

Cooking was sometimes seen as a meaningful and enjoyable thing to do. One woman liked to cook all the food, both for herself and for others. Four male respondents with food distribution appreciated it and felt it was easier to prepare the morning and evening meals, when they did not have to be responsible for the main meal. Several respondents chose to buy precooked frozen dishes and full meals for the main meal. One reason for this could be that they were frail and did not have enough strength to cook the main meal themselves. Another reason could be that it was boring to cook just for themselves. Prepared dishes were also considered easier to cook, for example, in a microwave, a cheap alternative and sometimes better tasting than the food that was served at the municipality's elderly centre.

* It is not fun to cook anymore, now when I'm alone, therefore I buy pre-cooked food (no. 7, female, age 84).*

* I buy pre-cooked food at the shop. I think it is better than the XXX (note, elderly centre restaurant)… (no. 7, female, age 84).*

* I do not miss cooking; it is enough with managing morning and evening meals (no. 9, male, age 89).*

#### 3.3.5. Eating

The issues that respondents raised about eating were, in most cases, related to their oral health and dental status. Broken teeth and poorly adapted prostheses could be the causes of difficulties with chewing. Some respondents thought the meat from food distribution was leathery and difficult to chew.

* Things that are hard to digest, such as brown beans, pea soup and stuff, I do not eat that, and whole meat, I cannot chew (no. 2, male, age 90).*

Problems with eating were also linked to the presence of other symptoms, such as fungal infection in the mouth and nausea caused by problems from the oesophagus and stomach. Difficulties in swallowing food were also connected to oral health, dental status, food content, and the cooking method. The respondents emphasised the importance of caring for their teeth and regular dental visits to maintain optimal dental and oral health.

* … It's worse now with the teeth. I can usually chew the food I get from… Yet, some of it is tough (no. 2, male, age 90).*

Most respondents had, at some time, choked on something. In two cases the situation had developed to a life-threatening condition. Items the respondents mentioned having choked on included tablets, crumbly bread, large pieces of food, and tough meat.

* … I'm a bit narrow in the throat… I choke… bread crumbs and things (no. 10, female, age 88).*

## 4. Discussion

The aim of the study was to describe home-living elderly people's views on important circumstances regarding food and meals. The analysis of the interviews showed that respondents' earlier life had a strong influence on current views of food and meals. Souter and Keller [31] presented similar results in a study describing how what older people ate depended on past life experiences and their approach to old age [31]. Other studies concerning food and meals showed that habits and preferences that are formed during childhood and youth are difficult to change in adult life [32] and that dietary intake was often similar to what the elderly people grew up with [33]. This has also been documented in other cultures. Interviews with elderly Taiwanese people showed only small changes in eating patterns, and how preferences for traditional habits from earlier generations influenced their food and meals [34]. Developing assistance regarding food and meals for elderly people requires knowledge about the individual's current needs, but habits founded in earlier life must also be taken into consideration.

The results of the study showed that the respondents' needs for help to manage their daily food and meals often arose in relation to a sudden life event. Stressful life events also contribute to an increased risk of developing malnutrition. Examples include being widowed or falling ill and requiring hospitalisation. They perceived their dependence on others both positively and negatively. Most respondents stated that the woman in the family had had the main responsibility for meals. Several men in the study reported that their wife's death had been the breaking point for becoming dependent on food distribution and they thought the food distribution was a good alternative to get nourishing food. However, women who previously had cooked all their food mentioned difficulties in accepting the situation and in reconciling themselves with the conditions of food distribution. This is consistent with a study that showed that being dependent on others was difficult to accept, but dependence on meals from food distribution could also mean better quality of life [35]. Another study showed that elderly women living alone tended to simplify cooking and eating and had fewer cooked meals and events with coffee with cakes [36], and that poor cooking skills among elderly men was a barrier to improving energy intake, healthy eating and appetite [37]. Sudden life events often change habits relating to food and meals, and men and women seem to adapt to the new circumstances in different ways depending on earlier roles and experiences. These events are important to capture in order to highlight the need for assistance so that necessary actions can be taken.

In this study habits founded in past life, and negative life events affected the food and meals in present life. It might be that also the view of the future influences current food intake. Shifflet has studied food habit changes in a couple of studies including elderly patients visiting nutrition sites [38, 39]. Even though the focus for these studies was on food habit changes there are some interesting implications for the understanding of the findings in the present study. In one of the studies [38], the temporal frameworks within which food habit changes are negotiated were explored. Food habit changes were found to be externally motivated (following physician-prescribed diets, altering food intake due to taste changes, social isolation, and reduced income) or internally motivated (self-prescribed diets, maintenance of lifelong food habits, reduction of food intake due to being less active). In addition, past experiences in conjunction with a negative or positive view of the future resulted in varying levels of compliance with special diets [38]. The respondents in the present study expressed a wish to maintain their lifelong food habits and it is likely that such a wish would negatively affect eventual efforts to achieve positive food habit changes, if that would be necessary. However, the respondents did not express any concerns about the future affecting their food habits to any great extent. This might be due to that they already had made some food habit changes in connection with previous negative life events. In another study by Shifflett and McIntosh [39] it was found that among people with a positive future time perspective 9.7% of respondents had made negative food habit changes. However, among those with a negative future time perspective 32.4% had made negative food habit changes. Some types of disruptions and concerns that may be associated with a negative view of the future and possible changes in food habits were identified—females, loss of spouse, living alone, perception of declining health, and low income [39]. Also in the present study especially loss of spouse, living alone, and declining health seemed to negatively influence the ability to acquire, prepare and/or consume food. Considering the findings from Shifflet's studies [38, 39] together with the findings from this study it seems important not only to consider the past experiences and negative life events but also the elderly persons future time perspective in the negotiation of appropriate food use patterns.

The results of the study are guided by the respondents' desires to choose and make decisions about shopping, cooking and eating. This could mean access to fresh food of good quality, sitting by yourself and eating in peace or the opportunity to go to the shop with the home care staff. Similar findings are also highlighted in a study by Pajalic et al. [35], where elderly people with food distribution wanted good food from natural products and tasting “home-cooked food” [35]. The findings showed that the quality of assistance offered to older people living in their own home is dependent on the ability to be involved in decisions about food and meals, and on whether the help is given on the individual's own terms and is designed based on personal preferences and habits [40]. Swedish health care laws [41] and social services [42] make it clear that care and help must be based on respect for individual autonomy and integrity. Ageing and disease can cause physical and mental decline, which may result in the individual no longer being able to make the choices he or she wishes, but having to adapt to the new situation. There is self-determination as long as the person can freely choose between offered options, even if it is not always the most wanted option [43]. For those with formal home help service, all care staff can help the older person to maintain their independence and autonomy by building trusting relationships, being aware of needs and offering necessary help. The older people's autonomy can be promoted and affirmed through empowerment and opportunities to influence their own situation. Writing shopping lists, having access to flyers, going shopping, cooking with staff, having more than one dish to choose from, and being able to influence the content of menus and dishes are factors that can affirm participation and empowerment.

In planning and implementing qualitative studies, it is important to consider factors such as trustworthiness and transferability. A prerequisite for forming an opinion on trustworthiness is that all steps in the research process are well described [30]. Data were collected through semistructured interviews with older people living in their own home. The respondents were recruited by the community unit heads and there is a risk that only those elderly who had positive feelings agreed to participate. We had no way of knowing how many declined to participate or why. The results of the study, however, reveal both positive and negative aspects, and despite a low number of respondents, the results can be considered to provide valuable insight into what is important to consider regarding food and meals for older people living in their own home. The interview texts were analysed using content analysis [30]. To minimise the risk of researcher influence, the two authors read the texts several times independently and then discussed their interpretation during each step of the analysis. To ensure that no data relating to the aim were excluded, the results were finally tested against the original texts [30]. The contents of the results are also supported by illustrative quotations that show extractions from the study interview texts [44].

## 5. Conclusions

Meeting the need for optimal nutritional status for older people living at home requires knowledge of individual preferences and habits, from both their earlier and current lives. It is important to pay attention to risk factors that could compromise an individual's ability to independently manage their diet, such as major life events and hospitalisation. Individual needs of self-determination and involvement should be considered in planning and development efforts for elderly people related to food and meals. Preventive home visits to elderly people, without home help service and/or meals from a formal agency, can be one way to capture difficulties with acquiring, preparing and/or consuming food. This can be done in order to give advice and/or suggest provision of formal help. Another intervention could be to develop a program, promoting eating with other seniors. Elderly people can be picked up at home and driven to an elderly centre restaurant at which they receive nutritious meals and opportunities to socialise with others.

## Figures and Tables

**Table 1:** Characteristics of the respondents.

Interview number	Gender	Age	Cohabitation	Living in town (T) Countryside (CS)	Home help service	Food distribution, days/week	Severity of disease^(1)^
1	Male	84	Alone	CS	No	7	Moderate
2	Male	90	Together	T	Yes	7	Mild
3	Female	87	Together	T	Yes	7	Severe
4	Female	94	Alone	T	Yes	7	Moderate
5	Male	82	Alone	T	No	7	None
6	Male	89	Alone	CS	Yes	3	Moderate
7	Female	84	Alone	T	Yes	5	Mild
8	Female	85	Alone	T	No	0	Moderate
9	Male	89	Alone	T	Yes	7	Mild
10	Female	88	Together	CS	No	0	Moderate
11	Male	94	Together	CS	No	0	Moderate
12	Male	86	Alone	T	No	7	Mild

^(1)^Self-perceived severity of disease graded as none, mild, moderate, or severe.

**Table 2:** Categories and subcategories regarding home-living elderly people's views of food and meals.

Categories	Subcategories
Habits founded in the past affect the present	Food and meals
	Roles

To get help from others with food and meals	The breaking point
	Transition from independence to dependence

	Meals during the day
	Quality of food
Food and meals in present life	Buying and transporting food
	Cooking
	Eating
